# Gut microbiota and SCFA signatures in predicting postoperative infection sites in neurocritical care patients

**DOI:** 10.3389/fmicb.2026.1933225

**Published:** 2026-09-03

**Authors:** Weirong Sun, Zhen Zhang, Congkai Wang, Junyao Huang, Demao Cao, Aijun Peng, Haixiao Jiang, Wei Zeng

**Affiliations:** 1 Department of Neurosurgery, Taizhou Second People’s Hospital Affiliated to Yangzhou University, Taizhou, China; 2 Department of Neurosurgery, Suzhou Hospital, Affiliated Hospital of Medical School, Nanjing University, Suzhou, China; 3 Department of Neurosurgery, Lianyungang Clinical College of Nanjing Medical University/The First People’s Hospital of Lianyungang, Lianyungang, China; 4 Department of Neurosurgery, Peking University People’s Hospital, Qingdao, Shandong, China; 5 Department of Neurosurgery, The Affiliated Hospital of Yangzhou University, Yangzhou, China; 6 Department of Neurosurgery, Yancheng First Hospital, Affiliated Hospital of Nanjing University Medical School, Yancheng, China

**Keywords:** gut microbiota, postoperative infection, predictive biomarker, risk stratification, short-chain fatty acids

## Abstract

**Background:**

Postoperative pulmonary infection (PI) and intracranial infection (IC) are major complications in neurocritical care patients; however, reliable approaches for predicting infection site susceptibility remain unavailable. Increasing evidence suggests that the gut microbiota and its metabolites influence systemic immune responses through the gut–lung and gut–brain axes. However, whether early postoperative gut microbial signatures could discriminate between subsequent PI and IC remains unknown.

**Methods:**

In this cross-sectional study, 90 neurocritical care patients undergoing neurosurgical procedures were enrolled and subsequently categorized based on their postoperative infection status into three groups: IC (*n* = 30), PI (*n* = 30), and non-infected controls (CON, *n* = 30). Fecal samples were collected within 24 h after surgery for 16S rRNA gene sequencing and gas chromatography–mass spectrometry-based quantification of short-chain fatty acids (SCFAs). Microbial diversity, taxonomic composition, differential microbial biomarkers, functional profiles, and microbiota–metabolite correlations were systematically analyzed.

**Results:**

Compared with CON patients, both infection groups exhibited significantly reduced microbial diversity and altered community structures. Distinct gut microbial and metabolic patterns were observed between IC and PI patients. The IC group showed enrichment in *Enterococcus, Staphylococcus*, and *Clostridium*, increased butyrate levels, reduced propionate levels, and elevated branched-chain SCFA proportions. In contrast, the PI group was characterized by expansion of *Proteobacteria*, enrichment of *Klebsiella* and *Escherichia-Shigella*, increased propionate concentrations, and decreased butyrate levels. Correlation analyses demonstrated strong associations between characteristic microbial genera and SCFA profiles. A six-genus microbial panel, comprising *Enterococcus, Faecalibacterium, Dialister, Escherichia-Shigella, Agathobacter*, and *Blautia*, effectively discriminated infection categories, achieving an area under the curve of 0.901. Functional prediction further revealed distinct metabolic and pathogenicity-related pathways among the three groups.

**Conclusion:**

Early postoperative gut microbiota and SCFA signatures are associated with subsequent infection sites in neurocritical care patients. These findings highlight the potential of gut microbial-metabolic profiling as a non-invasive strategy for early risk stratification and personalized prevention of postoperative pulmonary and intracranial infections.

## Introduction

Neurocritical care patients often require emergent surgical decompression or hematoma evacuation (Greenberg et al., 2022). Despite advances in perioperative management, postoperative infections remain a major determinant of clinical outcomes. Pulmonary infection (PI) and post-craniotomy intracranial infection (IC) are two of the most common complications, with reported incidences of approximately 40% and 0.7–8%, respectively (Battaglini et al., 2023; Carone et al., 2025; Li et al., 2020). These two infection types differ fundamentally in their clinical management. PI is predominantly caused by Gram-negative pathogens, such as *Klebsiella pneumoniae*, *Pseudomonas aeruginosa*, and *Acinetobacter baumannii*, and therefore requires antibiotics with adequate penetration into lung tissue (Almouwlid et al., 2025). Post-craniotomy meningitis is most frequently attributed to Gram-positive cocci, particularly coagulase-negative *Staphylococci* and *Staphylococcus aureus*, necessitating antibiotics that are capable of crossing the blood–brain barrier (Chang et al., 2018; Enrico et al., 2025). Preventive strategies also diverge: PI prevention emphasizes elevation of the head of the bed, subglottic suctioning, and oral care protocols, whereas IC prevention focuses on strict aseptic technique, external ventricular drain management, and appropriate perioperative antibiotic prophylaxis. Currently, no reliable tools exist to identify which neurocritical care patients are predisposed to developing pulmonary versus intracranial infection during the postoperative period. This knowledge gap precludes individualized preventive approaches and forces a uniform strategy for all patients.

The gut microbiota has emerged as a critical modulator of host immunity, influencing susceptibility to infections at distant sites (Zhang et al., 2024). Short-chain fatty acids (SCFAs)—primarily acetate, propionate, and butyrate—are key metabolites through which commensal bacteria regulate immune function (Li G. et al., 2025; Li S. et al., 2025; Li W. et al., 2025). SCFAs act via G-protein-coupled receptors (FFAR2 and FFAR3) on immune cells and through inhibition of histone deacetylases (HDACs), thereby reprogramming the epigenetic landscape of macrophages, neutrophils, and T cells (Maruyama et al., 2025; Zhao et al., 2026). The concepts of the gut–lung axis and gut–brain axis are well established, with accumulating evidence demonstrating that intestinal dysbiosis affects susceptibility to both respiratory and central nervous system infections (Li et al., 2024; Schneider et al., 2024; Ye et al., 2025). In critically ill patients, the gut microbiota undergoes profound dysbiosis characterized by depletion of SCFA-producing taxa (e.g., *Roseburia*) and expansion of pathobionts (e.g., *Enterobacteriaceae*; Wozniak et al., 2022; Xiao et al., 2025). Xu et al. (2019) demonstrated that neurocritical care patients exhibit significant reductions in butyrate-producing bacteria within 72 h of admission and that this dysbiosis independently predicts 180-day mortality. More recently, Zwicky et al. provided proof-of-concept that preoperative gut microbiota composition—specifically the *Firmicutes-to-Prevotella* ratio—predicts postoperative surgical site infections undergoing abdominal surgery patients (Zwicky et al., 2025). However, these studies have focused on whether infection occurs, not on where it occurs.

No study has investigated whether early postoperative gut microbial and metabolic signatures could discriminate between subsequent pulmonary and intracranial infections in neurocritical care patients. If baseline gut SCFA profiles differ systematically between patients who later develop PI and those who develop IC, fecal sampling could serve as a non-invasive risk stratification tool, enabling site-specific preventive strategies. Hence, in this study, we tested the hypothesis that early postoperative fecal SCFA and microbiota signatures differ among neurocritical care patients who subsequently develop PI, those who develop IC, and those who remain infection-free. We aimed to identify distinct microbial and metabolic fingerprints associated with each infection site to inform future personalized infection prevention strategies in neurocritical care.

## Materials and methods

### Participants and study design

**Figure 1:**
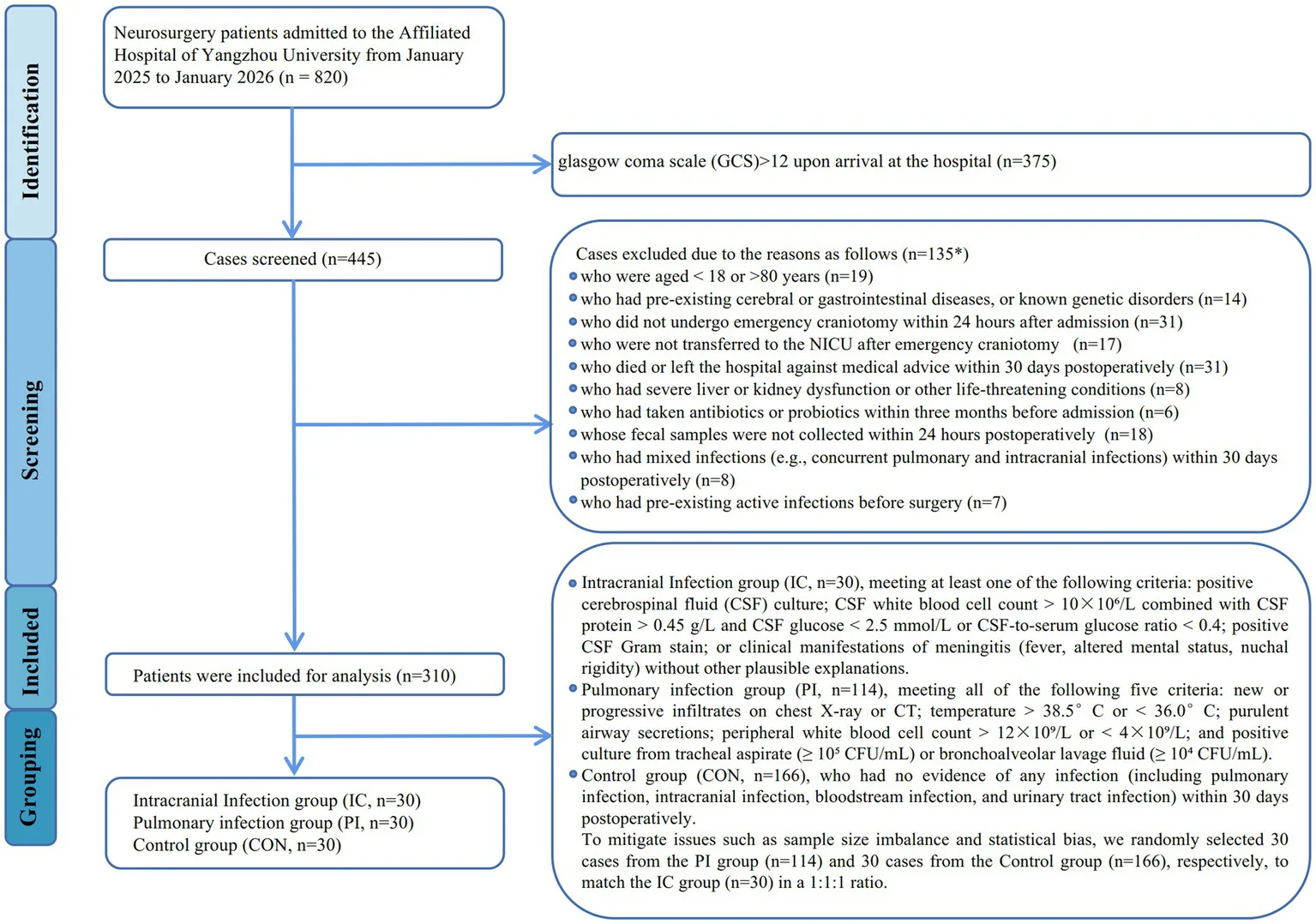
Flowchart shows the derivation of study participants. *Patients may be excluded for more than one factor.

This cross-sectional study was conducted at the Department of Neurosurgery, Yangzhou University, between January 2025 and January 2026. The study protocol was approved by the Institutional Ethics Committee (No. 2024-YKL09-K06). Written informed consent was obtained from all patients or their legal proxies, and all procedures were performed in accordance with the Declaration of Helsinki. Based on predefined inclusion and exclusion criteria, a total of 90 patients were enrolled, and the screening flowchart is shown in Figure 1.

### Fecal sample collection and processing

Fecal samples were collected 24-h post-surgery using sterile containers. Within 2 h of collection, samples were thoroughly homogenized, aliquoted into 2-mL cryovials, snap-frozen on dry ice, and then transferred to a −80 °C freezer for long-term storage.

### Short-chain fatty acid analysis

The concentrations of seven SCFAs—namely acetic, propionic, butyric, isobutyric, valeric, isovaleric, and caproic acids—were quantified in fecal samples utilizing gas chromatography–mass spectrometry (GC–MS). Briefly, 50 mg of feces were mixed with 500 μL of saturated sodium chloride solution and 20 μL of 10% sulfuric acid. After vortexing, the mixture was extracted twice with 800 μL of diethyl ether. The combined organic phases were evaporated under nitrogen, and the residue was derivatized with 50 μL of N-methyl-N-trimethylsilyltrifluoroacetamide (MSTFA, containing 1% trimethylchlorosilane) at 70 °C for 30 min. GC–MS analysis was performed on an Agilent 7890B-5977B system equipped with an HP-5MS capillary column (30 m × 0.25 mm × 0.25 μm). Helium was used as the carrier gas at a flow rate of 1.0 mL/min. The oven temperature program was as follows: an initial temperature of 80 °C with a hold of 1 min, followed by a ramp to 200 °C at 15 °C/min, then to 250 °C at 5 °C/min, and finally to 300 °C at 20 °C/min, with a final hold of 5 min. The injection volume was 1 μL and was performed in splitless mode. Mass spectra were acquired in full-scan mode (m/z 40–500) under electron impact ionization (70 eV). 2-Ethylbutyric acid was used as the internal standard, and calibration curves for the seven SCFAs were constructed, with all correlation coefficients (*R*^2^) exceeding 0.995. SCFA concentrations were expressed as μmol/g of wet feces.

### 16S rRNA gene sequencing and bioinformatics analysis

Total DNA was extracted from fecal samples using the QIAamp PowerFecal Pro DNA Kit, and DNA concentration and purity were assessed with a NanoDrop 2000. The V3–V4 hypervariable region of the 16S rRNA gene was amplified using primers 338F (5′-ACTCCTACGGGAGGCAGCA-3′) and 806R (5′-GGACTACHV GGGTWTCTAAT-3′). Amplicons were sequenced on the Illumina NovaSeq 6,000 platform (2 × 250 bp paired-end). Raw sequencing data were processed using QIIME2 (Licata et al., 2025) (version 2023.5). Quality filtering, denoising, and amplicon sequence variant (ASV) inference were performed using DADA2. Normalization was applied to the ASV sequence count table to mitigate discrepancies in sequencing depth, ensuring that each sample had an equal total sequence count and that all values were expressed as relative abundances normalized to 1. Taxonomic assignment was based on the SILVA 138 database. Alpha diversity indices were compared among the three groups to assess differences in richness and evenness (Jiang et al., 2025). Beta diversity was evaluated using Bray–Curtis dissimilarity and weighted UniFrac distances, visualized via principal coordinate analysis (PCoA) and non-metric multidimensional scaling (NMDS; Jiang et al., 2023; Komaki et al., 2024). Intergroup differences were validated by permutational multivariate analysis of variance (PERMANOVA; Adonis, 999 permutations) and analysis of similarities (ANOSIM, 999 permutations; Jiang et al., 2024). Differential genera were identified using linear discriminant analysis effect size (LEfSe), with a linear discriminant analysis (LDA) threshold of 2.0 and a *p-*value of <0.05(Khleborodova et al., 2024). Spearman’s rank correlation analysis was subsequently performed to examine associations between the relative abundances of differential genera and SCFA concentrations, with results visualized as heatmaps. For functional prediction, BugBase was used for phenotypic prediction, and PICRUSt2 was used to infer Kyoto Encyclopedia of Genes and Genomes (KEGG) pathway abundances from ASV sequences (Liu R. et al., 2025; Liu X. et al., 2025).

### Clinical data collection

Clinical data were extracted from the electronic medical record system. Demographic and baseline characteristics included age, sex, body mass index, smoking history, alcohol consumption, underlying comorbidities (including hypertension and type 2 diabetes mellitus), admission Glasgow Coma Scale (GCS) score, and primary disease type (traumatic brain injury/intracerebral hemorrhage). Infection-related parameters comprised peak body temperature within 24 h postoperatively, postoperative 24-h lactate, and white blood cell count. Surgical parameters included operative duration, intraoperative blood loss, and whether decompressive craniectomy was performed. Antibiotic use was recorded as the type of prophylactic antibiotic administered (cefuroxime/piperacillin).

### Statistical analysis

Statistical analyses were performed using SPSS 26.0 and R 4.2.0. Continuous variables are presented as mean ± standard deviation or median (interquartile range) and categorical variables as frequency (percentage). Comparisons among the three groups were conducted using the one-way analysis of variance or the Kruskal–Wallis test, with *post-hoc* pairwise comparisons performed using Tukey’s test or Dunn’s test with Bonferroni correction, as appropriate. Comparisons between two groups were carried out using the independent-samples t-test or the Mann–Whitney U-test, with *p*-values adjusted by Bonferroni correction for multiple comparisons. Categorical variables were compared using the chi-square test or Fisher’s exact test. All tests were two-tailed, and a *p*-value of <0.05 was considered statistically significant.

## Results

### Patient enrollment and baseline characteristics

**Figure 2:**
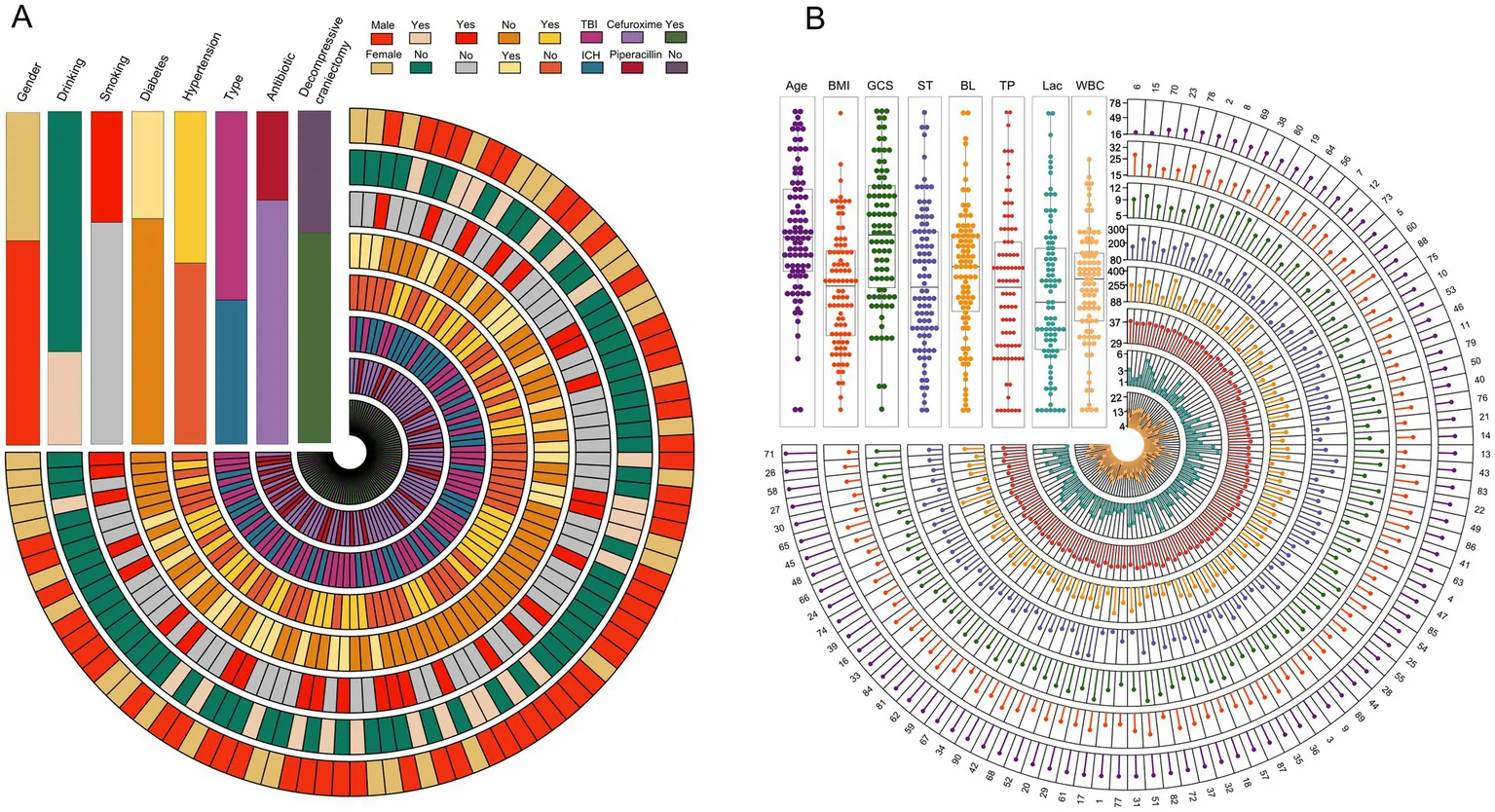
Demographics of all 90 participants. **(A)** indicates categorical data, and **(B)** indicates continuous data.

A total of 820 patients were screened, and 90 were ultimately enrolled and divided into three groups: the intracranial infection (IC, n = 30) group, the pulmonary infection (PI, *n* = 30) group, and the non-infected controls (CON, n = 30) group. The detailed screening and enrollment process are illustrated in Figure 1. No significant differences were observed among the three groups in age, sex, body mass index, or other baseline characteristics (all *p* > 0.05, Table 1), confirming baseline comparability across groups. Individual-level data for each participant are shown in Figure 2.

**Table 1:** Demographic characteristics of non-infected controls and patients with IC and PI.

Characteristics	CON (*n* = 30)	PI (*n* = 30)	IC (*n* = 30)	*p*-value (PI vs IC)	*p*-value (CON vs INF)
Demographics					
Age (years)	51.5 ± 14.2	56.3 ± 9.7	55.1 ± 12.0	0.656	0.124
Sex (male/female)	18/12	17/13	20/10	0.528	0.878
BMI (kg/m^2^)	23.3 ± 2.5	24.5 ± 2.4	23.9 ± 3.6	0.449	0.173
Smoking [*n*(%)]	10 (33.3)	9 (30.0)	11 (36.7)	0.861	0.590
Alcoholism [*n*(%)]	8 (26.7)	8 (26.7)	9 (30.0)	0.946	0.538
Comorbidities [*n*(%)]					
Hypertension	12 (40.0)	15 (50.0)	14 (46.7)	0.731	0.506
Type 2 diabetes	8 (26.7)	10 (33.3)	11 (36.7)	0.700	0.481
Diagnosis [*n*(%)]				0.873	0.660
TBI	16 (53.3)	18 (60.0)	17 (56.7)		
ICH	14 (46.7)	12 (40.0)	13 (43.3)		
Admission GCS	9.5 ± 1.4	9.3 ± 1.8	9.1 ± 1.1	0.711	0.380
Antibiotics [*n*(%)]				0.506	0.604
Cefuroxime	22 (73.3)	24 (80.0)	20 (66.7)		
Piperacillin	8 (26.7)	6 (20.0)	10 (33.3)		
Operative parameters					
Operative time (min)	175.5 ± 49.2	187.8 ± 45.0	198.0 ± 45.5	0.388	0.098
Blood loss (mL)	245.3 ± 65.0	242.7 ± 69.1	239.7 ± 58.8	0.857	0.772
Craniectomy [*n*(%)]	18 (60.0)	20 (66.7)	19 (63.3)	0.866	0.650
Postoperative parameters					
Peak temperature (°C)	36.8 ± 0.4	37.1 ± 0.7	36.9 ± 0.6	0.114	0.208
Lactate (mmol/L)	2.7 ± 1.2	2.8 ± 1.7	2.4 ± 1.2	0.269	0.738
WBC (×10^9^/L)	11.7 ± 3.2	12.4 ± 4.5	11.4 ± 3.3	0.349	0.878

Data are presented as mean ± SD or *n* (%). CON, control group; PI, pulmonary infection; IC, intracranial infection; INF, total infected patients. BMI, body mass index; TBI, traumatic brain injury; ICH, intracerebral hemorrhage; GCS, Glasgow Coma Scale; WBC, white blood cell.

### Fecal short-chain fatty acid profiles

**Figure 3:**
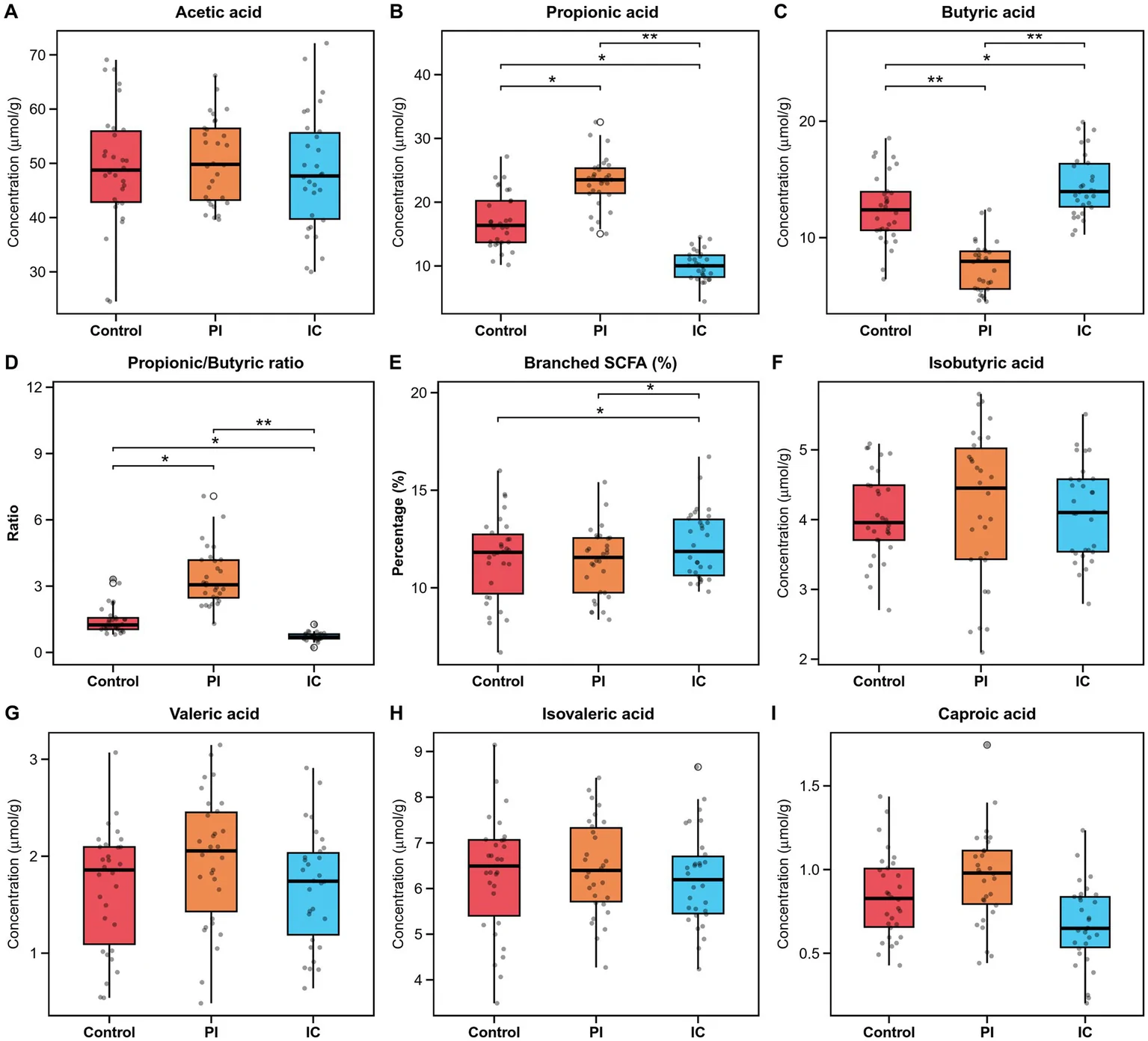
Concentrations of short-chain fatty acids (SCFAs) across three groups. Absolute concentrations of acetic, propionic, and butyric acids **(A–C)**. Propionic/butyric acid ratio **(D)**. Percentage of branched-chain SCFAs **(**sum of isobutyric and isovaleric acids, **E)**. Concentrations of isobutyric, valeric, isovaleric, and caproic acids **(F–I)**. Each dot suggests one sample. *p < 0.05 and **p < 0.01. CON, control group; PI, pulmonary infection; IC, intracranial infection.

Comparisons of fecal SCFA concentrations among the three groups are shown in Figure 3. Compared with the CON group, the PI group exhibited elevated propionate, reduced butyrate, and consequently an increased propionate/butyrate ratio. In contrast, the IC group showed reduced propionate, elevated butyrate, and a decreased propionate/butyrate ratio. The proportion of branched-chain SCFAs (sum of isobutyrate and isovalerate) to total SCFAs was significantly higher in the IC group than in the PI and CON groups (*p* < 0.05). No significant differences were detected among groups in acetate, isobutyrate, valerate, isovalerate, or caproate concentrations (all *p* > 0.05).

### Gut microbiota diversity was reduced in both the IC and PI groups

**Figure 4:**
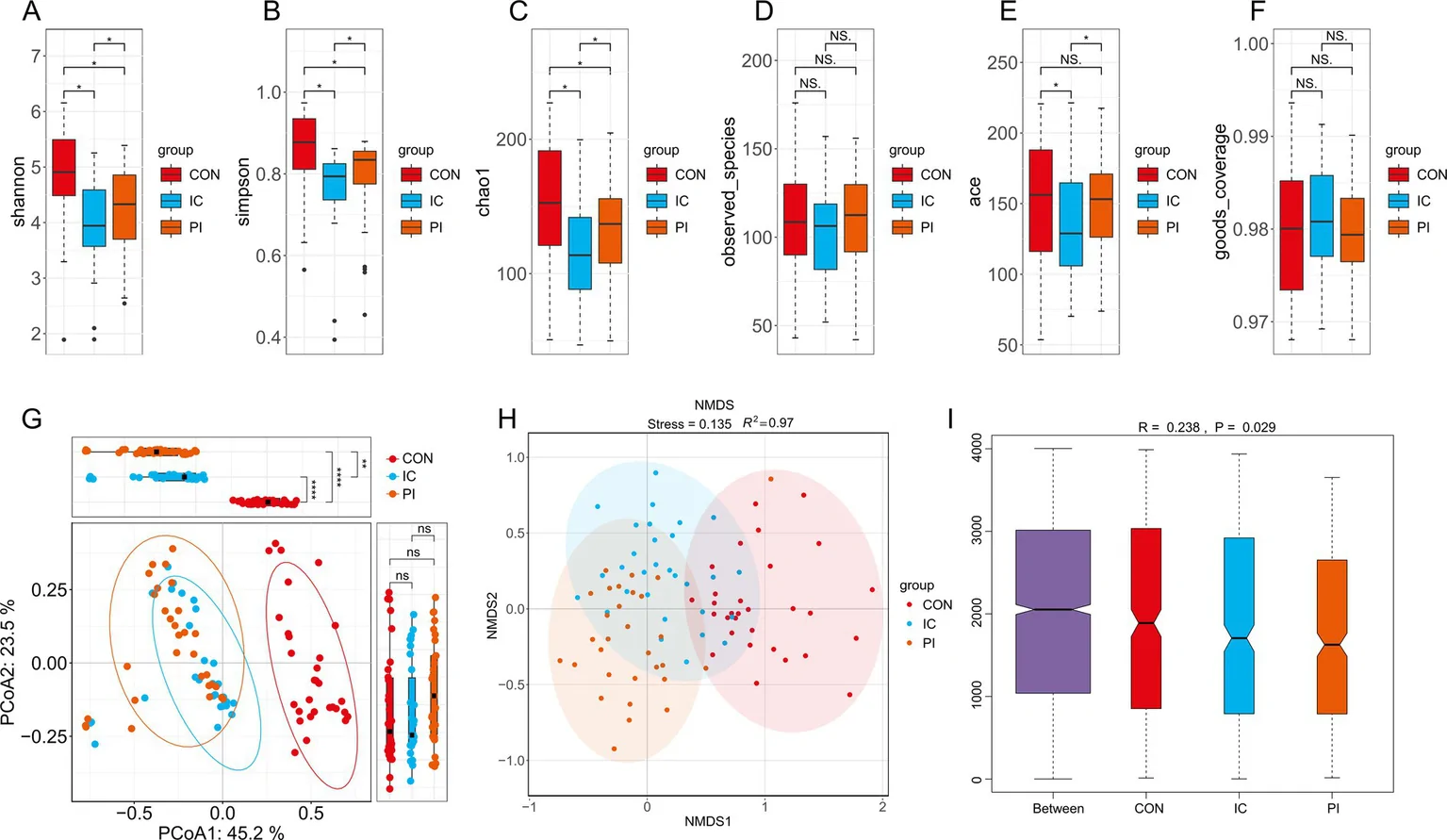
Comparison of gut microbial diversity and community structure among the Control, PI, and IC groups. Alpha diversity indices, including Shannon index, Simpson index, Chao1, Observed species, ACE, and goods coverage, show within-group diversity **(A–F)**. Beta diversity analysis based on principal coordinate analysis (PCoA) and non-metric multidimensional scaling (NMDS) using Bray–Curtis distance **(G–H)**. Each dot represents an individual sample, with closer distances indicating more similar microbial community compositions. Analysis of similarities (ANOSIM) shows the *R*-statistic and corresponding *p*-value to test for significant differences between groups **(I)**. CON, control group; PI, pulmonary infection; IC, intracranial infection.

To explore whether microbial community diversity differed among the three groups, alpha diversity analysis was performed (Figures 4A–F). As shown in Figures 4A–C, the Shannon, Simpson, and Chao1 indices all differed significantly among the three groups. This finding revealed that both infection groups had significantly lower diversity than the CON group across all three indices, and the IC group had significantly lower values than the PI group (*p* < 0.05). These three indices collectively reflect both species richness and evenness, indicating marked differences in microbial community diversity among the three groups. For the other alpha diversity indices (Figures 4D–F), the Abundance-based Coverage Estimator (ACE) index (Figure 4E) also showed significant intergroup differences, while the remaining two indices (Figures 4D,F) did not.

For beta diversity, principal coordinate analysis (PCoA) based on Bray–Curtis distances revealed three distinct clusters corresponding to the three groups. The CON group separated from the two infection groups, suggesting that the infection status was associated with systematic alterations in microbial community structure. Separation was also observed between the PI and IC groups, indicating that the infection site exerted an independent effect on microbial composition (Figure 4G, *R*^2^ = 0.18, *p* < 0.001). Non-metric multidimensional scaling (NMDS) analysis yielded a consistent separation pattern (stress = 0.135 < 0.2, Figure 4H), and ANOSIM further supported that inter-group differences exceeded intra-group variability (R = 0.238, *p* = 0.029, Figure 4I). Collectively, these multidimensional analyses have corroborated that differences in microbial composition are not only between infected and non-infected patients but also between patients with different infection sites.

### Gut microbiota composition and intergroup differences

**Figure 5:**
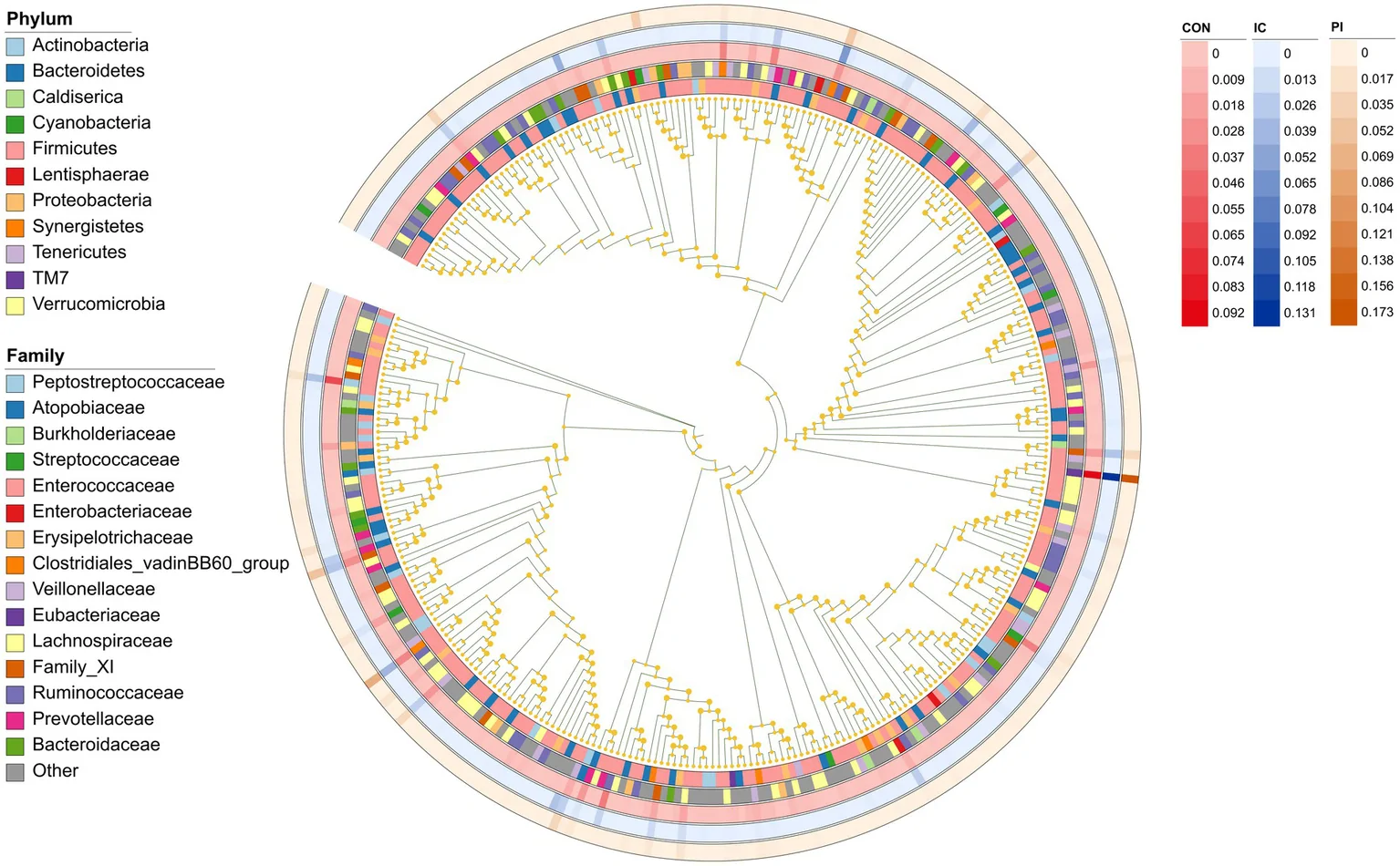
Circular phylogenetic tree shows the taxonomic landscape of the gut microbiota. The internal branching structure illustrates evolutionary relationships inferred from 16S rRNA gene sequences, with each terminal node representing an individual amplicon sequence variant (ASV) grouped by sequence homology. Surrounding the tree are five layered annotation rings (from inner to outer): the first two rings denote the phylum- and family-level taxonomic assignments for each ASV; the outer three rings visualize the relative abundance profiles of ASVs across the CON (red), IC (blue), and PI (orange) groups, where color intensity reflects abundance magnitude. CON, control group; PI, pulmonary infection; IC, intracranial infection; ASV, amplicon sequence variant.

A total of 12,843,720 high-quality sequences were obtained, with an average of 142,708 sequences per sample (range: 112,345–168,932). Based on this dataset, 1,346 amplicon sequence variants (ASVs) were identified, belonging to 11 phyla, 60 families, and 319 genera (Figure 5). To further characterize compositional differences in the gut microbial community among the CON, PI, and IC groups, we systematically compared relative abundances across taxonomic levels.

**Figure 6:**
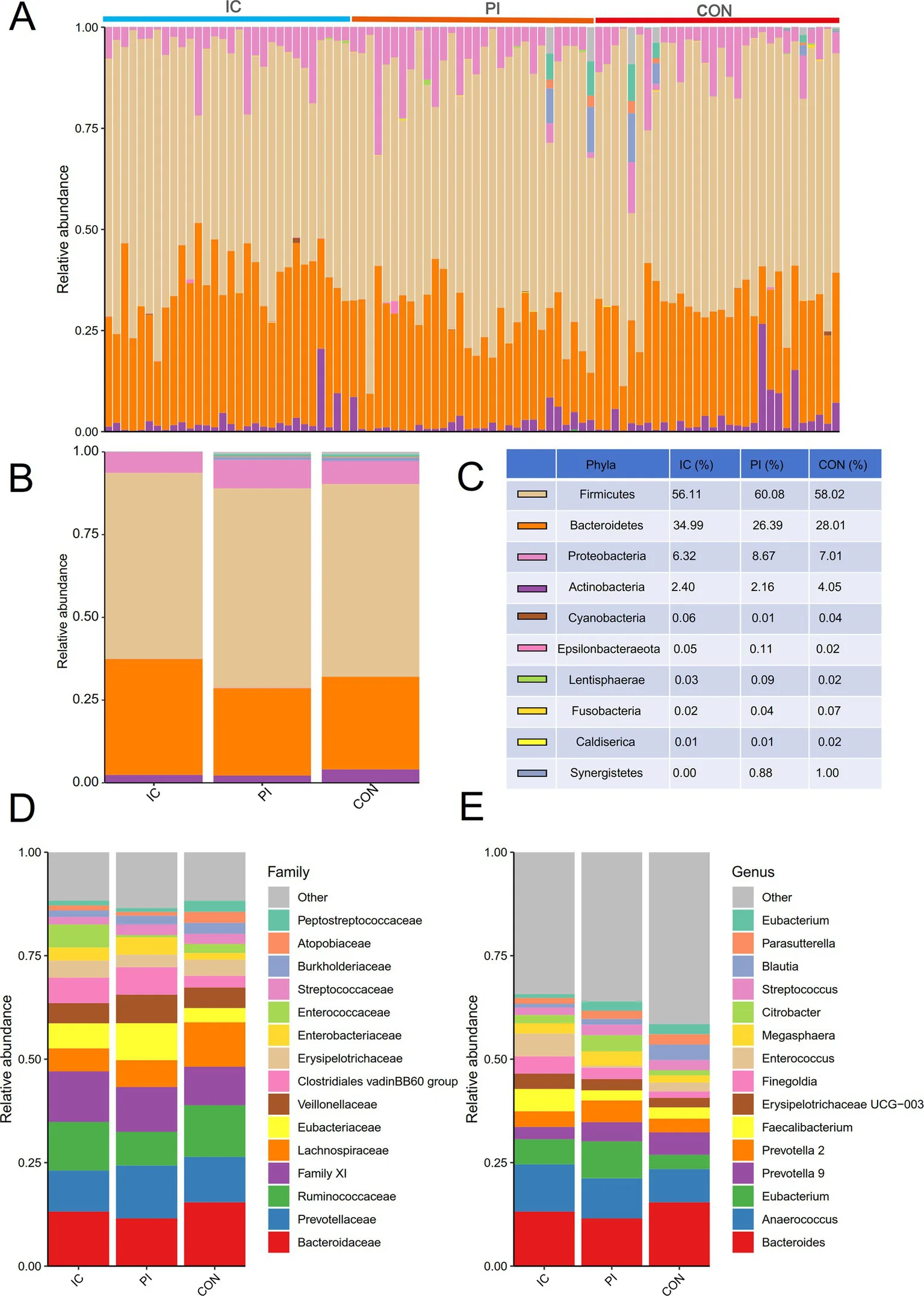
Gut microbiota composition analysis across different groups. Stacked bar plot shows the relative abundance of bacterial phyla in all individual samples. Each bar represents an individual sample, grouped by IC (blue), PI (orange), and CON (red) cohorts **(A)**. Bar plot displays the average relative abundance at the phylum level for the three groups **(B)**. Table details the relative abundance percentages of the predominant phyla across the IC, PI, and CON groups **(C)**. Stacked bar plot shows the average relative abundance at the family level **(D)** and at the genus level **(E)** across the three groups. CON, control group; PI, pulmonary infection; IC, intracranial infection.

Taxonomic profiling at the phylum level revealed that *Firmicutes* and *Bacteroidetes* were the dominant bacterial lineages across all three groups, collectively accounting for 86.03, 86.47, and 91.10% of the total sequences in the CON, PI, and IC groups, respectively. Among the three groups, the IC group exhibited the lowest relative abundance of *Firmicutes* (56.11%), whereas the PI group showed the highest proportion (60.08%), followed by the CON group (58.02%). Conversely, the relative abundance of *Bacteroidetes* was lowest in the PI group (26.39%) and highest in the IC group (34.99%), with the CON group falling in between (28.01%). *Proteobacteria*, the third most abundant phylum, was abundant in the PI group (8.67%) than in the CON (7.01%) and IC (6.32%) groups. *Actinobacteria* showed the highest relative abundance in the CON group (4.05%), approximately twofold higher than that observed in the PI (2.16%) and IC (2.40%) groups. The remaining phyla, including *Cyanobacteria*, *Epsilonbacteraeota*, *Lentisphaerae*, *Fusobacteria*, and *Caldiserica*, were present in relatively low abundances (<0.1%) across all treatment groups (Figures 6A–C).

At the family level, the top five abundant families varied considerably among the three groups. In the CON group, the predominant families were *Bacteroidaceae, Ruminococcaceae, Prevotellaceae, Lachnospiraceae*, and *Family XI*. The PI group was dominated by *Prevotellaceae, Bacteroidaceae, Family XI, Eubacteriaceae*, and *Ruminococcaceae*. In the IC group, the most abundant families included *Bacteroidaceae, Family XI, Ruminococcaceae, Prevotellaceae*, and *Clostridiales vadinBB60 group*. Notably, *Lachnospiraceae* was enriched in the CON group but absent from the top five in both infection groups, whereas *Eubacteriaceae* emerged as a dominant family, particularly in the PI group. Furthermore, *Enterobacteriaceae* exhibited an expansion in the PI group, while *Enterococcaceae* showed an enrichment in the IC group. Meanwhile, inter-group variations became even more intricate at the genus level (Figures 6D–E).

### Characteristic microbial genera among three groups

**Figure 7:**
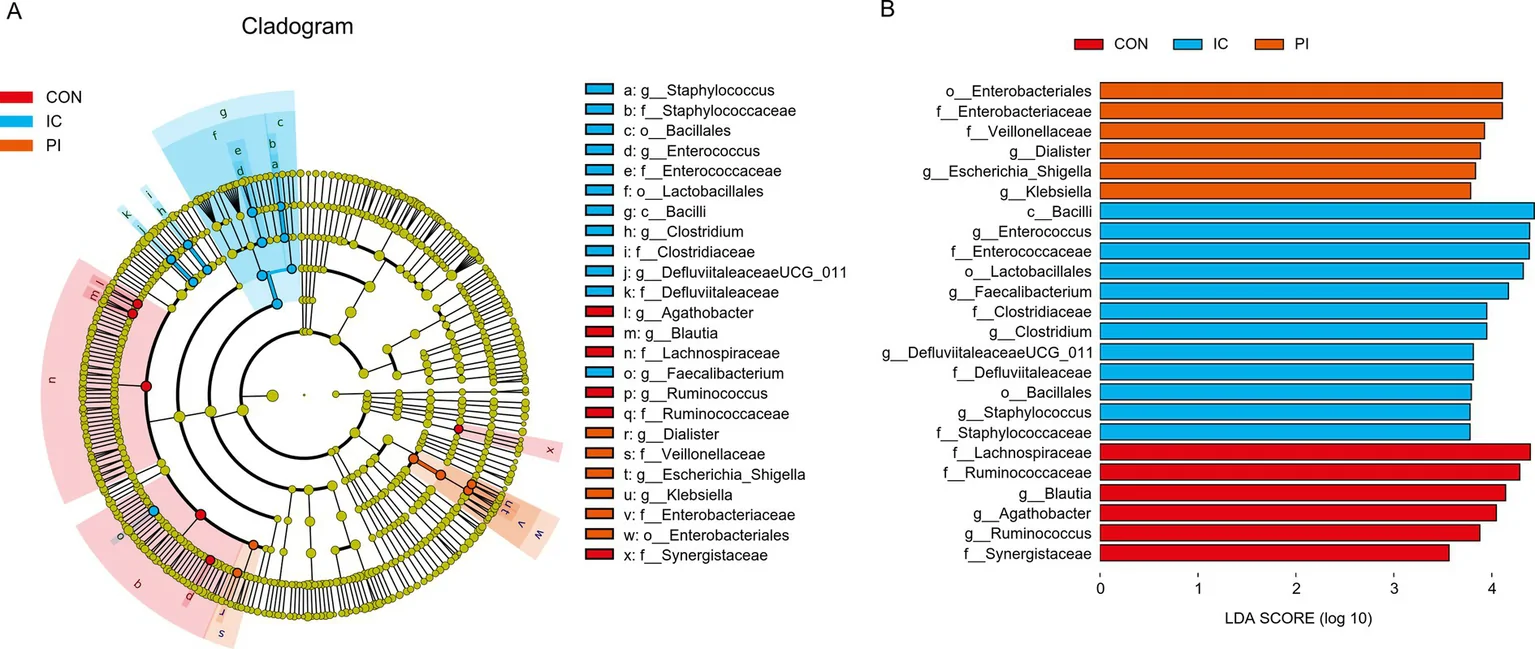
LEfSe analysis identifies differentially abundant bacterial taxa among the CON, IC, and PI groups. Cladogram illustrates the phylogenetic distribution of bacterial lineages significantly associated with the three groups **(A)**. Nodes colored in red, blue, and orange represent bacterial taxa that are significantly enriched in the CON, IC, and PI groups, respectively. Yellow nodes indicate taxa with no significant difference. The letters correspond to the specific taxonomic names listed in the right-hand legend (prefixes c_, o_, f_, and g_ represent class, order, family, and genus, respectively). Histogram of LDA scores shows the effect size of differentially abundant taxa. Higher LDA scores (X-axis) indicate a stronger contribution of the taxa to the differentiation between groups. Colors represent the group in which each taxon is most abundant **(B)**. LEfSe, linear discriminant analysis effect size; LDA, linear discriminant analysis; CON, control group; PI, pulmonary infection; IC, intracranial infection; ASV, amplicon sequence variant.

To identify differentially abundant microbial taxa among the three groups, we performed LEfSe analysis (linear discriminant analysis effect size) with an LDA threshold of >2.0 and *p* < 0.05. As shown in Figure 7, a total of 24 microbial genera were identified as characteristic of the three groups, among which 6 were enriched in the PI group, 12 in the IC group, and 6 in the CON group. The PI group was characterized by the enrichment of opportunistic pathogens including *Klebsiella* and *Escherichia/Shigella*, as well as the propionate-producing genus *Dialister*. The IC group was predominantly characterized by the opportunistic pathogens *Enterococcus*, *Staphylococcus*, and *Clostridium*, as well as the butyrate-producing genus *Faecalibacterium*. In contrast, the CON group was characterized by the butyrate-producing genera *Blautia, Agathobacter*, and *Ruminococcus*.

### A panel of microbial markers for distinguishing PI and IC from CON

**Figure 8:**
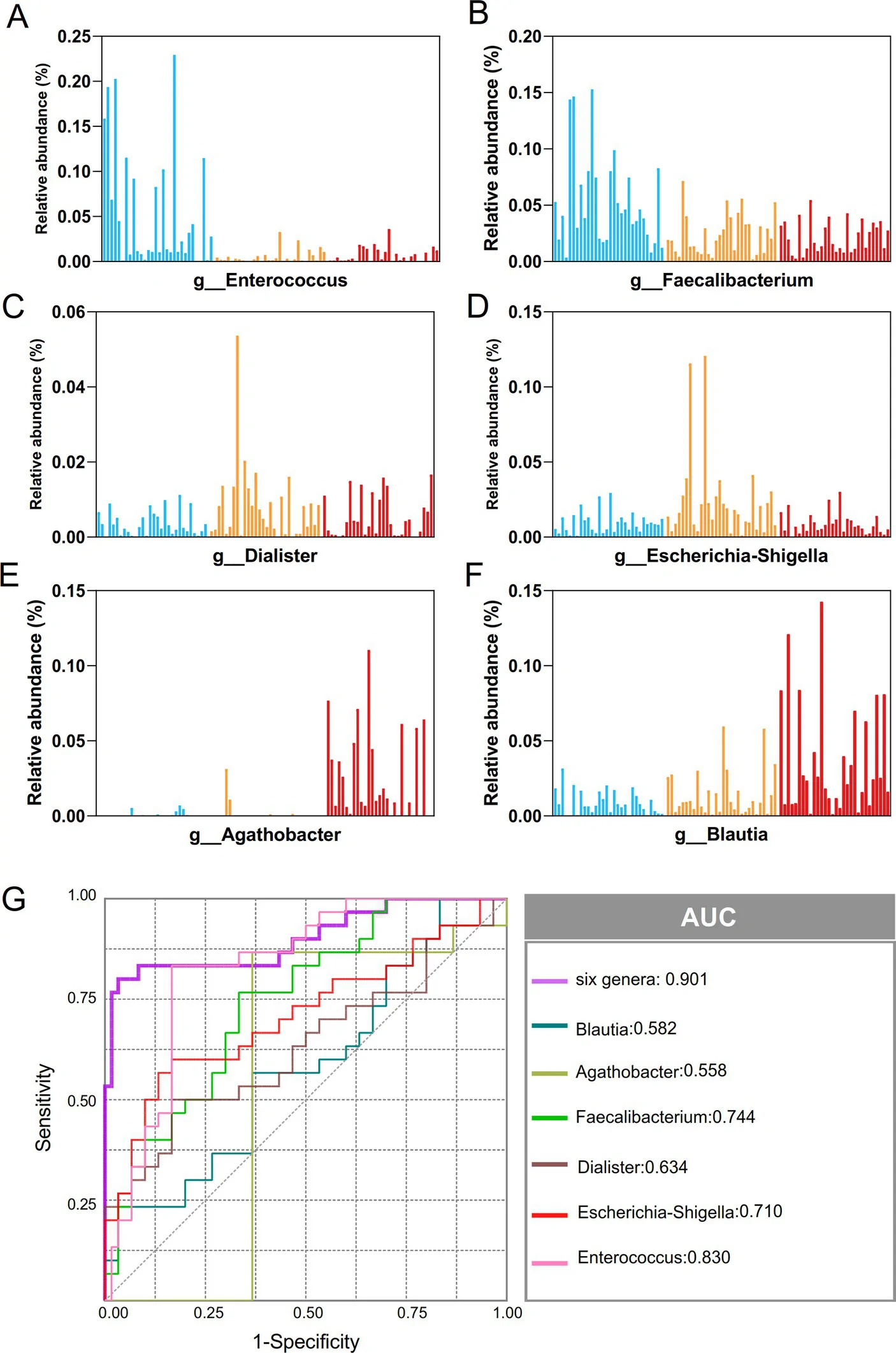
Relative abundance distribution of specific genera and their diagnostic performance. Bar plots show the relative abundance (%) of six representative genera (*Enterococcus*, *Faecalibacterium*, *Dialister*, *Escherichia–Shigella*, *Agathobacter*, and *Blautia*) across individual samples in three groups **(A–F)**. Bars are color-coded to indicate the corresponding group. Receiver operating characteristic (ROC) curve analysis evaluates the predictive power of these genera for group discrimination. The area under the curve (AUC) values are provided in the legend **(G)**. CON, control group; PI, pulmonary infection; IC, intracranial infection.

This study identified multiple gut microbial genera associated with PI and IC, including *Enterococcus, Faecalibacterium, Dialister, Escherichia/Shigella, Agathobacter*, and *Blautia*, which were frequently detected across the CON, PI and IC groups, with marked interindividual variation in their abundances (Figures 8A–F). We further evaluated the predictive potential of these six genera, alone or in combination, for subsequent infection types. The ROC analysis demonstrated that the combined panel yielded substantially improved predictive performance, with an AUC of 0.901 (Figure 8G). These findings suggest a strong association between the microbial signature and subsequent infection categories, supporting the reliability and utility of this predictive strategy.

### Altered microbial phenotypic characteristics in patients with PI and IC

**Figure 9:**
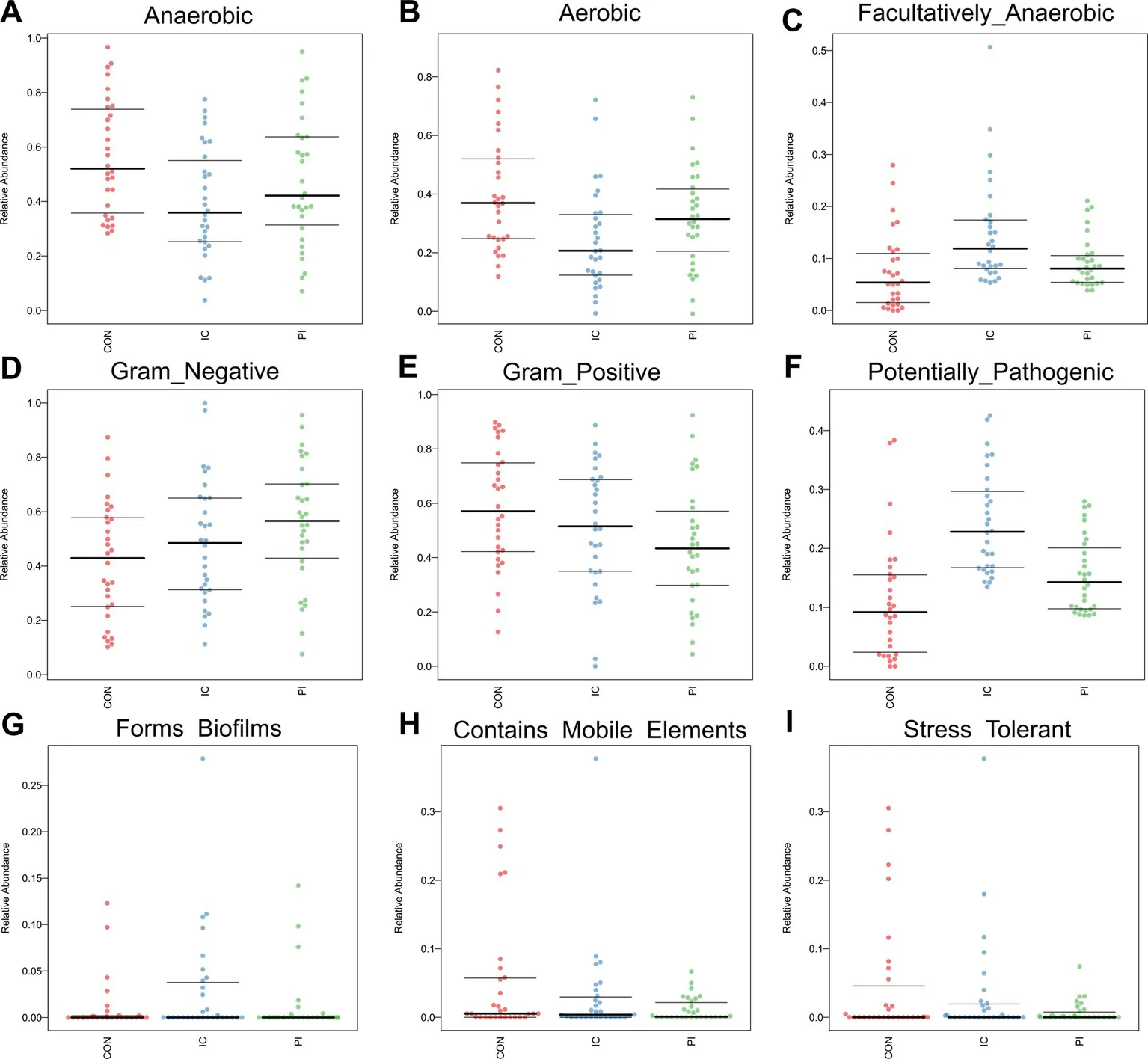
Comparison of predicted microbial phenotypic functions among the CON, IC, and PI groups. Scatter plots display the relative abundance of various predicted bacterial phenotypes across the three groups: **(A)** anaerobic, **(B)** aerobic, and **(C)** facultatively anaerobic; **(D)** Gram-negative, **(E)** Gram-positive, **(F)** and potentially pathogenic; **(G)** forms biofilms, **(H)** contains mobile elements, and **(I)** stress-tolerant. Each dot represents an individual sample.

BugBase analysis was performed to predict the phenotypic characteristics of gut microbial communities across the three groups. Compared with the CON group, both the PI and IC groups exhibited significantly lower relative abundances of aerobic bacteria (*p* = 0.032), anaerobic bacteria (*p* = 0.016), and Gram-positive bacteria (*p* = 0.018). Conversely, the relative abundances of Gram-negative bacteria (*p* = 0.018), potentially pathogenic taxa (*p* = 0.036), and facultatively anaerobic bacteria (*p* = 0.021) were significantly higher in both infection groups. No significant differences were observed among the three groups in biofilm-forming capacity (*p* = 0.66), mobile element content (*p* = 0.19), or oxidative stress tolerance (*p* = 0.32) (Figures 9A–I).

### Correlation between characteristic gut microbiota and SCFA metabolites

**Figure 10:**
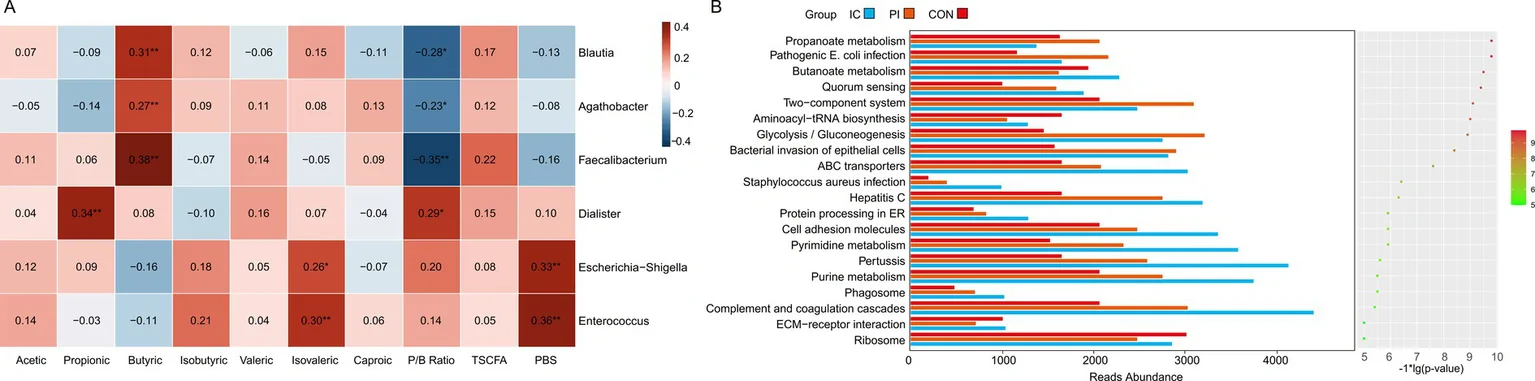
Correlation analysis between specific genera and SCFAs, and comparison of predicted functional pathways among the three groups. Heatmap displays the Spearman’s correlation coefficients between the relative abundance of six key genera and SCFA parameters. Red indicates a positive correlation, and blue indicates a negative correlation. *p < 0.05 and **p < 0.01 **(A)**. Combined bar plot and scatter plot depict the relative abundance and differential significance of KEGG pathways across the CON, IC, and PI groups. The left panel shows the read abundance of each pathway for the three groups. The right panel displays the statistical significance as −log10 (*p*-value), with dot colors indicating the magnitude of the significance **(B)**. CON, control group; PI, pulmonary infection; IC, intracranial infection; P/B ratio, *Prevotella*-to-*Bacteroides* ratio; TSCFAs, total short-chain fatty acids; PBS, percentage of branched short-chain fatty acids; KEGG, Kyoto Encyclopedia of Genes and Genomes.

Spearman’s correlation analysis was performed between six characteristic genera and nine SCFA parameters (Figure 10A). All butyrate-producing genera exhibited significant positive correlations with butyric acid concentration, with *Faecalibacterium* showing the strongest association (*r* = 0.38, *p* = 0.001), followed by *Blautia* (*r* = 0.31, *p* = 0.002) and *Agathobacter* (*r* = 0.27, *p* = 0.008), and all three were significantly negatively correlated with the propionic/butyric acid ratio (*p* < 0.05) (Montanari et al., 2022; Van-Wehle and Vital, 2024). *Dialister*, a signature genus enriched in the PI group, showed a significant positive correlation with propionic acid concentration (*r* = 0.34, *p* = 0.004), as well as with the propionic/butyric acid ratio (*r* = 0.29, *p* = 0.011), which was highly consistent with the metabolic features of elevated propionic acid and an increased propionic/butyric acid ratio observed in the PI group. The opportunistic pathogens *Escherichia–Shigella* and *Enterococcus* were both significantly positively correlated with branched-chain SCFA parameters. The former was associated with isovaleric acid (*r* = 0.26, *p* = 0.018) and branched-chain SCFA percentage (*r* = 0.33, *p* = 0.002), while the latter was correlated with isovaleric acid (*r* = 0.30, *p* = 0.005) and branched-chain SCFA percentage (*r* = 0.36, *p* = 0.001).

### PICRUSt2 functional prediction

Gut microbiota dysbiosis could trigger systemic metabolic disturbances and subsequently reshape the structure and function of the microbial community (Wang et al., 2025). To evaluate differences in gut microbial function among the three groups, we used PICRUSt2 to predict Kyoto Encyclopedia of Genes and Genomes (KEGG) pathway abundances from 16S rRNA sequencing data and identified the 20 most differentially abundant pathways across groups (Figure 10B). The PI group showed enrichment in propanoate metabolism and in pathways associated with pathogenic *Escherichia coli* infection, consistent with its characteristic enrichment of *Enterobacteriaceae* and active propionate metabolism. The IC group exhibited enrichment of butanoate metabolism, *Staphylococcus aureus* infection, quorum sensing, and ABC transporter pathways. The enrichment of butanoate metabolism in the IC group corresponded to the elevated abundance of *Faecalibacterium*, while the presence of quorum sensing and ABC transporter pathways suggested that pathobionts in this group may regulate virulence and environmental adaptation through these mechanisms. In contrast, the CON group displayed the highest abundance of aminoacyl-tRNA biosynthesis and ribosome pathways, reflecting active protein synthesis and metabolic stability. These findings indicate that gut microbial functions differ systematically among the three groups. The directional differences in functional dysbiosis may influence host susceptibility to distinct types of infection.

## Discussion

Postoperative infections remain among the most prevalent complications in neurosurgical critically ill patients, with intracranial and pulmonary infections demonstrating persistently high incidence rates and significantly affecting neurological recovery and long-term prognosis (Li G. et al., 2025; Li S. et al., 2025; Li W. et al., 2025). In recent years, the gut microbiota and its metabolites have garnered increasing attention as crucial immunomodulatory entities in the pathogenesis of infectious diseases (Wan et al., 2022). Unlike previous studies that primarily focused on the mere presence or absence of infection, the current study explores the differences in infection sites and, for the first time, introduces the concepts of the “gut-lung axis” and “gut-brain axis” within the context of postoperative infections in neurocritically ill patients. We analyzed baseline fecal samples to identify gut microbiota and short-chain fatty acid profiles that differentiate infection sites among patients who later developed postoperative pulmonary or intracranial infections, or remained infection-free. Our results showed that those who developed infections had lower microbial diversity, fewer beneficial bacteria, and more opportunistic pathogens, with distinct dysbiotic patterns that were associated with the subsequent infection type. Intracranial infections (IC) were characterized by a significant reduction in microbial diversity, an overrepresentation of *Enterococcus* and *Staphylococcus*, increased levels of butyrate, and decreased levels of propionate. In contrast, pulmonary infections (PI) were characterized by an expansion of *Proteobacteria*, an enrichment of *Klebsiella* and *Escherichia/Shigella*, elevated propionate levels, and reduced butyrate levels. Notably, correlation analyses between key genera and short-chain fatty acid (SCFA) metabolites further confirmed that the coordinated disruption of the microbiota–metabolite axis was closely associated with the specificity of the site of infection. The study reveals a connection between the initial gut microbiome and the sites of postoperative infections, with each site exhibiting distinct microbial imbalances. This insight may aid in evaluating risk and tailoring prevention strategies for postoperative infections in neurocritically ill patients.

### Depletion of microbial diversity and predilection for infection sites

In the current study, patients diagnosed with either intracranial or pulmonary infections exhibited significantly reduced gut microbial alpha diversity, as measured by the Shannon, Simpson, and Chao1 indices, compared with non-infected controls (CON). Notably, the IC group exhibited the lowest diversity. From a susceptibility standpoint, decreased gut microbial diversity serves as a non-specific marker of host susceptibility (Wozniak et al., 2024). This reduction in diversity suggests an increased number of unoccupied ecological niches and diminished colonization resistance, which, in turn, facilitate the enteric proliferation of pathogenic microorganisms and their potential dissemination to extra-intestinal locations (Cole et al., 2025; Wozniak et al., 2024). Meanwhile, the substantial disparity in diversity between the IC and PI groups indicates that the degree of diversity depletion may influence the tendency for specific infection sites. We propose that the more pronounced loss of diversity observed in the IC group may be associated with significant thinning of the intestinal mucus layer and increased epithelial permeability (Qu et al., 2021). This condition potentially facilitates the translocation of enteric bacteria and their products across the intestinal barrier, allowing entry into the portal circulation and systemic bloodstream and ultimately potentially crossing the blood–brain barrier to induce central nervous system infection (Granados-Martinez et al., 2024). This hypothesis aligns with clinical observations suggesting that intracranial infections are more prevalent in cases involving severe neurological injuries and prolonged durations in intensive care units.

### Baseline microbiota–metabolite ecotypes and the divergence of infection sites

The LEfSe analysis, SCFA quantification, and correlation analyses collectively identified two distinct baseline “microbiota-metabolite” configurations with divergent trajectories. This suggests that varying gut ecological contexts may influence distinct susceptibility profiles.

The first configuration, predictive of intracranial infection risk, was microbiologically underpinned by an over-specialized butyrate production pathway. In the IC group, *Faecalibacterium* served as the dominant butyrate-producing bacterium in baseline feces (Shi et al., 2025), whereas the relative abundances of other butyrate-producing genera, including *Blautia, Agathobacter*, and *Ruminococcus*, were significantly reduced (Dicks, 2024; Montanari et al., 2022), indicating a severe deficiency in community redundancy within the butyrate metabolic pathway. Concurrently, opportunistic pathogenic genera such as *Enterococcus*, *Staphylococcus*, and *Clostridium* were co-enriched, constituting a potential pathogen reservoir (Tozzo et al., 2022). At the metabolic level, this group exhibited the highest baseline butyrate concentration and the lowest propionate concentration among the three groups, resulting in the lowest propionate/butyrate ratio. Nonetheless, increased concentrations of butyrate do not necessarily convey a protective signal. The immunomodulatory effects of butyrate exhibit a non-monotonic, bell-shaped dose–response relationship. Moderate concentrations of butyrate confer protective effects by promoting the differentiation of regulatory T cells (Tregs), maintaining the integrity of the intestinal barrier, and suppressing excessive inflammatory responses (Li G. et al., 2025; Li S. et al., 2025; Li W. et al., 2025; Parada-Venegas et al., 2025). However, when butyrate production transitions from a synergistic output involving multiple taxa to dominance by a single genus and surpasses a certain threshold, its effects may become detrimental (McBride et al., 2023; Peng et al., 2007; Wang et al., 2023). Moreover, excessively high butyrate concentrations may over-suppress Th17 cell and neutrophil functions, potentially weakening the host’s initial defense against invasive pathogens and thereby facilitating bloodstream-derived bacterial colonization in the central nervous system (Wen et al., 2021). Furthermore, as a histone deacetylase inhibitor, butyrate at excessively high concentrations may downregulate the expression of blood–brain barrier tight junction proteins (e.g., claudin-5 and ZO-1), thereby increasing baseline permeability of the central nervous system (Sittipo et al., 2022). In addition, a high-butyrate environment may inhibit the growth of propionate-producing bacteria through negative feedback, thereby reducing the balancing effect of propionate as an immunomodulatory buffer (Flint et al., 2024; Shetty et al., 2022). Meanwhile, the IC group exhibited a significantly elevated proportion of branched-chain short-chain fatty acids, indicative of enhanced intestinal protein fermentation (Nikolaki et al., 2023), which is often accompanied by the production of potentially deleterious byproducts, such as ammonia and phenolic compounds, that may impair mitochondrial function in the intestinal epithelium and compromise barrier integrity (Gilbert et al., 2018). The expansion of *Enterococcus* and *Staphylococcus* constituted the microbial basis for pathogen translocation. The coexistence of these two conditions in the IC group indicates a highly vulnerable intestinal barrier, providing favorable conditions for bacterial translocation. Functional prediction analysis further revealed that the IC group exhibited enrichment of pathways associated with *Staphylococcus aureus* infection and quorum sensing at baseline. This suggests that opportunistic pathogens may have already been in a state conducive to the activation of virulence genes prior to the onset of infection, with quorum sensing facilitating the synchronized initiation of bacterial attacks (Ziegert et al., 2024). Additionally, enrichment of the “ABC transporters” pathway was associated with enhanced drug efflux pump activity (How et al., 2024). Collectively, the synergistic interaction among hyperactive butyrate metabolism, quorum sensing activation, and compromised barrier integrity suggests that the central defense system of patients in this group may be significantly weakened, thereby potentially predisposing them to pathogenic bacterial colonization of the central nervous system and the progression to IC.

The second configuration, predictive of PI risk, exhibited distinct microbial and metabolic features, characterized by the synergistic enrichment of *Klebsiella*, *Escherichia/Shigella*, and *Dialister*, along with elevated baseline propionate concentrations and decreased butyrate concentrations. *Klebsiella* and *Escherichia/Shigella*, members of the *Enterobacteriaceae* family of opportunistic pathogens, possess lipopolysaccharide (LPS) in their cell walls, which serves as a potent agonist of Toll-like receptor 4 (TLR4) (Ma et al., 2025; Wei et al., 2025). The substantial colonization of these microorganisms in the intestine may result in a continuous source of low-level endotoxin production. Lipopolysaccharides (LPS) originating from the gut can enter the systemic circulation through the mesenteric lymphatic pathway and the portal venous system, potentially leading to chronic exposure of alveolar macrophages to circulating LPS (Dey, 2025; Liu et al., 2021). This exposure might sustain TLR4 signaling in a state of persistent low-grade “pre-activation” (Liu et al., 2021). The pre-activation of macrophages, in isolation, does not initiate infection. However, it may modify the pulmonary response to subsequent bacterial exposure. Upon inhalation of bacteria, these pre-activated macrophages could release substantial quantities of pro-inflammatory cytokines, such as TNF-α, IL-1β, and IL-6 (Hoogerwerf et al., 2010). Nonetheless, heightened inflammatory responses do not necessarily correlate with efficient bacterial clearance. Elevated levels of pro-inflammatory cytokines may inhibit macrophage phagocytic activity, compromise the integrity of the alveolar epithelial barrier, and potentially delay the activation of specific Th1 immune responses (Baloglu et al., 2022; Liu R. et al., 2025; Liu X. et al., 2025; Pesce et al., 2022). More critically, sustained LPS exposure may, following the inflammatory peak, induce a state of “endotoxin tolerance” or “immunoparalysis” in macrophages, characterized by diminished antigen-presenting capacity and impaired bactericidal activity (Guo et al., 2026; Moerings et al., 2025). Consequently, the core dilemma faced by the lungs in the PI group was the coexistence of excessive inflammatory responses and inadequate pathogen-elimination efficiency. Meanwhile, *Dialister*, as a propionate-producing genus, may, via GPR41 signaling, enhance hematopoietic stem cell differentiation toward macrophages and dendritic cells, resulting in an increased baseline abundance and hyperreactivity of pulmonary antigen-presenting cells (APCs) (Trompette et al., 2014). It should be emphasized that propionate’s impact on the immune system is complex: Under normal conditions, moderate levels support immune tolerance through GPR41/GPR43 (Kim, 2023). However, during lung infections, elevated propionate levels, combined with LPS-induced inflammation and low butyrate, may enhance inflammation rather than protect against it. Under such conditions, APCs may enter a state of “signal saturation” due to persistent activation, such that, when bacterial infection occurs, their responsiveness to additional stimulation by bacterial antigens diminishes, impairing their ability to upregulate MHC-II and co-stimulatory molecules to activate naive T cells (Kim et al., 2019). Furthermore, Tregs represent a critical force in restraining APC hyperactivation; the reduced butyrate levels in the PI group may weaken Treg differentiation and induction, thereby compromising negative regulation of APCs (He et al., 2024; Tekguc et al., 2021). Collectively, excessive inflammatory activation, inefficient pathogen clearance, and aberrantly elevated propionate and insufficient butyrate increase susceptibility to pulmonary bacterial infection. The correlation analysis confirmed a positive association between *Dialister* abundance and propionate concentration (r = 0.34), and PICRUSt2 functional prediction also revealed enrichment of “propionate metabolism” and “pathogenic *Escherichia coli* infection” pathways in this group, functionally corroborating the above features.

The non-infected control group presented a distinctly protective microbial landscape, characterized by the synergistic enrichment of *Blautia*, *Agathobacter*, and *Ruminococcus*. In this group, butyrate-producing bacteria exhibited a multi-genus coexistence pattern with a balanced community structure, without any single genus overdominating. At the metabolic level, both butyrate and propionate concentrations remained at moderate levels, with an intermediate propionate/butyrate ratio and the lowest proportion of branched-chain short-chain fatty acids among the three groups. PICRUSt2 functional prediction revealed enrichment of “aminoacyl-tRNA biosynthesis” and “ribosome” pathways in this group, reflecting active protein synthesis and metabolic resilience (Tennakoon and Cui, 2024). The high-redundancy, multi-source microbial network represents a genuinely protective ecological configuration. In the event that one genus experiences external perturbation, other members might swiftly compensate, thereby potentially preserving the stability of intestinal barrier function and immunomodulatory output. This dynamic may confer robust resistance against infections in both intracranial and pulmonary regions.

### Clinical translational implications and study limitations

From a clinical translational perspective, the present study has several important implications for early identification, risk stratification, and microbiome-based interventions for postoperative infections in neurosurgical settings. First, alterations in gut microbial diversity and in the abundance of specific genera may serve as early-warning biomarkers of the onset of infection. Dynamic monitoring of postoperative gut microbiota trajectories holds the potential as an adjunctive tool for early infection screening. Second, based on the distinct microbiome signatures associated with different infection sites, precision probiotic/prebiotic intervention strategies could be formulated. For instance, in patients at high risk of PI, supplementation with butyrate-producing bacteria to elevate intestinal butyrate levels may enhance intestinal barrier function and mitigate pulmonary inflammation via the gut–lung axis. However, for patients at risk of IC, butyrate supplementation strategies warrant cautious evaluation, as indiscriminate butyrate enhancement may produce counterproductive effects. Third, the enrichment of quorum sensing and ABC transporter pathways suggests that drug efflux mechanisms should be adequately considered in the anti-infective therapy, and conventional antibiotic regimens may need to be combined with quorum sensing inhibitors or efflux pump inhibitors to improve therapeutic efficacy.

Several limitations of this study should be acknowledged. First, while our study design measured microbial signatures at a single early post-operative time point (24 h) that temporally precedes the clinical diagnosis of infection—which typically occurs days to weeks later—we acknowledge that a single measurement cannot distinguish whether these microbial features reflect pre-existing characteristics, acute responses to surgical stress and perioperative interventions, or a combination of both. Moreover, despite our efforts to control for key clinical confounders through strict inclusion criteria and baseline comparisons, we cannot completely exclude the possibility of residual confounding from unmeasured factors inherent to observational studies. Therefore, although our findings support the potential utility of early post-operative gut microbiota and SCFA profiles as early warning indicators for subsequent infections, they should be interpreted as hypothesis-generating rather than as definitive proof of mechanistic causality. Future prospective studies with longitudinal sampling—including pre-operative baseline, early post-operative, and follow-up time points—are warranted to further validate the predictive value of these microbial signatures and to elucidate the temporal dynamics of microbiota–host interactions in this critically ill population. Second, the sample size was relatively limited, with only 30 cases per group, which constrained the ability to conduct stratified analyses of additional potential confounding factors. Third, this was a single-center study, and the generalizability of our findings needs to be validated through multicenter studies with larger sample sizes. Fourth, PICRUSt2 functional predictions were indirect inferences based on 16S rRNA gene data rather than direct measurements of gut microbial metabolic activity. Fifth, we did not measure SCFA concentrations in peripheral blood or cerebrospinal fluid. Therefore, whether gut-derived SCFAs indeed enter the circulation and reach the target organs—a prerequisite for validating the proposed gut–lung and gut–brain axes—remains to be determined in future studies.

## Conclusion

Early postoperative gut microbiota and SCFA profiles were distinctly associated with subsequent pulmonary and intracranial infections in neurocritical care patients. Different infection sites exhibited unique microbiota–metabolite patterns, with PI characterized by enrichment of *Proteobacteria, Klebsiella*, and *Escherichia/Shigella*, accompanied by increased propionate levels, whereas IC was associated with reduced microbial diversity, enrichment of *Enterococcus* and *Staphylococcus*, and increased butyrate levels. A combination of characteristic microbial genera demonstrated the potential value in discriminating infection categories. These findings suggest that early gut microbiome and metabolite profiling may serve as a non-invasive approach to postoperative infection risk stratification and provide a basis for developing personalized prevention strategies in neurocritical care patients.

## Data Availability

The raw data supporting the conclusions of this article will be made available by the authors, without undue reservation.
